# SARS-CoV-2-associated organs failure and inflammation: a focus on the role of cellular and viral microRNAs

**DOI:** 10.1186/s12985-023-02152-6

**Published:** 2023-08-09

**Authors:** Reyhaneh Rasizadeh, Parisa Shiri Aghbash, Javid Sadri Nahand, Taher Entezari-Maleki, Hossein Bannazadeh Baghi

**Affiliations:** 1 Immunology Research Center, Tabriz University of Medical Sciences, Tabriz, Iran; 2 Infectious and Tropical Diseases Research Center, Tabriz University of Medical Sciences, Tabriz, 5166/15731 Iran; 3 Drug Applied Research Center, Tabriz University of Medical Sciences, Tabriz, Iran; 4 Department of Virology, Faculty of Medicine, Tabriz University of Medical Sciences, Tabriz, Iran; 5 Cardiovascular Research Center, Tabriz University of Medical Sciences, Tabriz, Iran; 6 Department of Clinical Pharmacy, Faculty of Pharmacy, Tabriz University of Medical Sciences, Tabriz, Iran

**Keywords:** SARS-CoV-2, COVID-19, microRNA, Inflammation, Organ failure

## Abstract

SARS-CoV-2 has been responsible for the recent pandemic all over the world, which has caused many complications. One of the hallmarks of SARS-CoV-2 infection is an induced immune dysregulation, in some cases resulting in cytokine storm syndrome, acute respiratory distress syndrome and many organs such as lungs, brain, and heart that are affected during the SARS-CoV-2 infection. Several physiological parameters are altered as a result of infection and cytokine storm. Among them, microRNAs (miRNAs) might reflect this poor condition since they play a significant role in immune cellular performance including inflammatory responses. Both host and viral-encoded miRNAs are crucial for the successful infection of SARS-CoV-2. For instance, dysregulation of miRNAs that modulate multiple genes expressed in COVID-19 patients with comorbidities (e.g., type 2 diabetes, and cerebrovascular disorders) could affect the severity of the disease. Therefore, altered expression levels of circulating miRNAs might be helpful to diagnose this illness and forecast whether a COVID-19 patient could develop a severe state of the disease. Moreover, a number of miRNAs could inhibit the expression of proteins, such as ACE2, TMPRSS2, spike, and Nsp12, involved in the life cycle of SARS-CoV-2. Accordingly, miRNAs represent potential biomarkers and therapeutic targets for this devastating viral disease. In the current study, we investigated modifications in miRNA expression and their influence on COVID-19 disease recovery, which may be employed as a therapy strategy to minimize COVID-19-related disorders.

## Introduction

Severe acute respiratory syndrome coronavirus 2 (SARS-CoV-2) is responsible for the recent Coronavirus disease 2019 (COVID-19) pandemic [1], and the global health worldwide has been significantly endangered by it [2]. SARS-CoV-2 infection has been estimated to have caused 544 million infections and 6.34 million fatalities in 228 countries as of March 2022 [3]. The virus belongs to *Coronaviridae* family and *Nidovirales* order, which are enveloped, positive sensed, single stranded RNA viruses causing diseases like COVID-19 and common cold. SARS-CoV-2 consists of multiple structural proteins including spike glycoprotein (S), envelope (E), membrane (M) and nucleocapsid (N). S protein is responsible for the attachment of virus to host receptor. The receptor of SARS-CoV-2 is angiotensin-converting enzyme 2 (ACE2) which is expressed in multiple organs, thus effecting multiple organs and causing organ failure i.e., lung, kidney, heart, brain, intestine, and liver [4–8]. The symptoms vary from mild to severe pneumonia [5, 9]. It has been discovered that the virus is related to acute respiratory distress syndrome, acute lung injury, and chronic obstructive pulmonary disease [5]. In addition, symptoms including fever, dry cough, diarrhea, muscle aches (myalgia), pneumonia, malaise, sore throat, shortness of breath and also neurological manifestations such as encephalopathy and encephalomyelitis, ischemic stroke and intracerebral hemorrhage, anosmia, as well as inflammatory diseases, have also been reported in COVID-19 infection [10–13]. Following the importance of the disease, many therapies have been developed, such as antibiotics and antiviral drugs, glucocorticoid/steroid therapy, invasive and non-invasive ventilation, and vaccination [14–16]. Given to this fact that these therapeutic strategies are not unique, safe, and definitive treatments and have different side effects in different people, and also the efficiency of vaccines may vary among different viral variants [17–19]; miRNAs as a biomarker can be an alternative treatment strategy in COVID-19 infection. Last two decades, small non-coding RNAs, such as miRNAs, have received much attention due to their potential role in regulating gene expression as well as the diagnosis of human diseases [20–22]. miRNAs are small endogenous non-coding RNAs that can regulate gene expression. MiRNAs are encoded by both viral genome and human host cell. Viral miRNAs (vmiRNA) end in viral replication promotion and host cell miRNAs may have anti-viral activities or even supporting viral evasion [17, 23–26]. miRNAs not only bind to the 3′ untranslated region of messenger RNAs (mRNAs) to restrict them, which is degradation of mRNA or translation inhibition, but also can bind to 5′ untranslated region (UTR), gene promoters, and coding sequences [5, 27].

Furthermore, these non-coding RNAs are involved in many physiological and pathophysiological functions in the cell including innate and adaptive immunity regulation, proliferation, and autophagy [27–29]. In fact, miRNAs stimulate the antiviral immune response by inducing T lymphocytes. Changes in miRNA levels in patients with respiratory disorders, diabetes, heart, and kidney failure are associated with the severity of COVID-19 [17, 23] (Table 1). What is more, miRNAs have important functions in host antiviral responses in many viral infections as well, including herpes virus, polyomavirus, retroviruses, pestivirus, hepacivirus, and COVID-19 [27, 30]. Likewise, miRNAs can have regulatory actions via multiple pathways in COVID-19 cases [5], and these small molecules might have significant roles in angiotensin-converting enzyme 2 (ACE2) and transmembrane serine protease 2 (TMPRSS2) regulation, which are the receptor and co-receptor of the virus respectively and are responsible for entry and membrane fusion of the virus, thus being a good choice to be inhibited as a therapy. For instance, 67 human miRNAs can target S glycoprotein gene and some viral miRNAs are capable of targeting host cell mRNAs and promoting viral replication i.e. a viral miRNA can suppress RNA polymerase II in the host cell [27]. Nevertheless, sufficient evidence is not available on the effect of viral miRNAs and host miRNAs on the cellular response to the virus. In this paper, we review the human and viral miRNAs involved in COVID-19 infection, as well as their influence and function on SARS-CoV-2’s target organs, with the goal of improving COVID-19 treatment strategies. The aim of this review is to be aware of miRNAs’ roles as potential therapeutic options for COVID-19 patients.

**Table 1:** Some of the important cellular and viral miRNAs’ role in SARS-CoV-2 targeted organs

Viral target	miRNA	miRNA Type	Function	References
Immune system and Inflammation	MR385-3p	Viral miRNA	Binding to 5′-UTR TGFBR3 as a major immunosuppressant	[31]
	MR147-5p		Binding to two inflammatory proteins such as CXCL16 and ARRB2	
	MR66-3p		Binding to TNF-activator	
	MR198-3p		Binding to IFN-related genes	
	MR359-5p and MR328-5		Binding to proteins associated with viral infection such as MYH9 and RARA	
	MR147-3p		Binding to TMPRSS2 enhancer	
	miR-146a	Host miRNA	Targeting the IRAK1 and TRAF6 proteins, suppressing IFN-α production	[32]
	miR-766-3p		Suppress IL-6 secretion following TNF-α production	[33]
	miR-423-5p, miR-23a-3p, and miR-195-5p		Produced in early stage of infection	[34]
	hsa-miR-939-5p and hsa-miR-146b-3p		Maintain homeostasis by reducing inflammation	[31]
Lung	MD241-3P	Viral miRNA	Inhibiting morphogenetic protein receptor type 2(BMPR2) gene	[27]
	MR288-5p and MD202-5p		Inflammation enhancement	[27]
	MD-2-5p and MR-147-3p		Reducing host cell apoptosis	[27]
	miR-155	Host miRNA	Related to more severe inflammatory symptoms	[35]
	miR-320 family		Affecting Nrf 2 expression, TGF-β signaling pathways, Hippo signaling pathways, and inflammation	[36]
	miR-200b-3p, miR-200c-3p, miR-429		Downregulating ACE2 expression	[5]
	miR-143-3p		Inhibit ACE2 expression	[37]
	miR-27b		Influence the expression of ACE2 receptor	[5]
	hsa-miR-98-5p		Bind to 3′ UTR of TMPRSS2 gene transcription	[27]
	hsa-let-7e/hsa-mir-125a and hsa-mir-141/hsa-miR-200		Suppress ACE2/TMPRSS2 gene transcription	[27]
			Preventing SARS-CoV-2 genome replication by regulating BCL2	[38]
	miR-1307-3p		Influence TGF-β gene and consequently play a key role in lung fibrosis	[39]
	hsa-miR-939-5p and hsa-miR-146b-3p		Maintain hemostasis by reducing inflammation	[40]
Heart	miR-26b-5p and miR-200c-3p	Host miRNA	Binding to the 3′-UTR region of ACE2 mRNA in cardiomyocytes	[41–43]
	miR-200c		Reduces the expression of ACE2 in cardiomyocytes	[41]
	miR-98-5p		Inhibit TMPRSS2 expression	[44]
	miR-98		Regulating TMPRSS2 function	[44]
	miR-21-5p, miR-155-5p and miR-214		Modulate the pro-inflammatory cytokine release in the heart	[44, 45]
	miR-125b and miR-223-3p		Modulate the insulin signaling pathway and heart function regulate IL-6 and TNF-α production	[46]
	miR-590-3p		Leads to myocarditis and heart failure	[47]
	miR-146a		Decreases inflammation and cardiac injury and Reduces sepsis-induced cardiac failure or diabetes mellitus	[48]
	miR-30e-3p		Suppresses virus replication	[23]
			Increase neutrophil count MPO promotion Increase endothelial cell apoptosis	[49]
	miR-133a		MMPs promotion	[50–52]
	miR-423-5p miR-23a-3p and miR-195-5p		Increase COVID-19 infection severity in CVD patients	[53, 54]
	miR-208a and miR-375		Promote apoptosis in ischemic cardiomyocytes	[55–57]
Brain	CvmiR-5-5p	Viral miRNA	Critical components in neurological diseases	[20]
	mir-155	Host miRNA	Regulate the SOCS1, SH2-SHIP1, C/EBP, and IL-13R	[58–61]
			Increases differentiation and inflammatory responses Inducing neuroinflammation	[62]
	mir-146a		Regulate STAT-1, IRF-5, and CFH Altered polarization of macrophages and microglia	[63–66]
	mir-124		Leads to an anti-inflammatory response through the M2 phenotype	[67–69]
	mir-21		Reduces TNF-α secretion in macrophages and microglia Increases IL-10 expression	[70, 71]
	mir-27b		Targeting an anti-inflammatory transcriptional activator PPAR Induces the secretion of inflammatory cytokines such as IL-6 and TNF-α	[72]
	mir-326		Affects the Th17 cells differentiation	[73]
	let-7		Activate inflammatory signaling pathways	[74]
Gastrointestinal	miR-147-3p	Viral miRNA	Targets the TMPRSS2 enhancer Increases intestinal inflammation Increases virus invasion to intestinal cells	[31]
	hsa-miR-4661-3p	Host miRNA	Affected to the S gene of SARS-CoV-2	[31]
	miR-21		Inhibit ACE2 gene expression	[5, 75]
	let-7a/ let-7d, miR-30a, miR-30c, miR-127, miR-194, miR-200c, miR-361, and miR-423		Inhibit the expression of TMPRSS2	[5, 75]
	miR-31-5p		Modulates inflammation	[34]
			Suppresses the expression of STAT6, SOCS1, CCL26	[76]
			Limit the excessive inflammatory response	[34]
	miR-3529-5p, miR-7-1-3p, and miR-548az-5p		Related to some of the COVID-19 symptoms	[6]
	miR-20b, miR-19a, miR-19b-3p, and miR-106a		Cleaving the SARS-CoV-2 S protein via regulating furin isoforms	[77]
Kidney	miR-141, miR-200a, let-7, miR-216a		Overexpression of TGF-β, Kidney fibrosis	[23]
	miR-181a		Upregulate the signaling cascades of adaptive immunity Decrease COVID-19 renal symptoms	[23, 78]
	miR-18 and miR-125b		Causes kidney disorder	[36]
Liver	MR198-3p	Viral miRNA	Suppressing IFN system responses	[79]
	miR-122	Host miRNA	Affecting innate immune response by RTKs/STAT3 signaling pathway, Production of type I and II IFNs,	[80]
			Affecting the amount of plasma or serum proteins	[81]

## Biogenesis and function of miRNAs

Small RNAs are RNAs with the length of 20–30 nucleotides. There are many small RNAs identified in cells, which are classified into three main groups including miRNA, siRNA and PIWI-interacting RNA (piRNA). MiRNAs are small (~ 22 nucleotides) non-coding RNAs that play a major role in RNA silencing and one third of human genes are regulated by miRNAs [82, 83]. They can affect most protein-coding transcripts and degrade mRNAs, thus playing a key role in every biological and pathological processes. MiRNA genes are one of the most plentiful gene families and they are produced not only in animals, but also in plants, protists and viruses [82, 83]. Scientists have been making consistent efforts towards devising techniques for detecting miRNAs, which involve a meticulous integration of experimental protocols and bioinformatics. The process of miRNA identification has gained momentum in recent times, primarily owing to the rapid progress of next-generation sequencing methodologies and in silico approaches [84]. MiRBase, miRDB, VIRmiRNA, and Starbase are a few of the existing publicly accessible miRNA databases. After the initial miRNA naming standard, mature miRNAs are distinguished by a numeric identifier following the prefix “miR”. To differentiate between animal and plant miRNAs, a dash symbol is used in animal miRNAs. However, it is important to note that this distinction is not made in the initial miRNA naming convention. [85]. Moreover, pre-miRNAs from animals are denoted using lowercase, for example, “mir-14.” However, plant pre-miRNAs are named differently [86]. A common identification number is used to refer to the same miRNA in multiple organisms, but with a three-letter prefix to denote the species name and a dash before the identifier number, for example hsa-miR-19b which is a *Homo sapiens* miRNA [85].

The biogenesis of miRNAs, which occurs in two main steps and in each step a ribonuclease III protein (RNase III) is involved, is extremely controlled and any dysregulation can be related to human diseases [82, 83]. The majority of miRNAs are recognized to be produced through a canonical biogenesis pathway. MiRNAs’ processing enzymes in humans are Drosha and Dicer [83]. First, Drosha cleaves primary miRNA (pri-miRNA) in nucleus, which was transcribed by RNA polymerase II, to release an ~ 70 nucleotide stem loop which is named the precursor miRNA (pre-miRNA). Following recognition of the pri-miRNA, the RNA binding protein Dgcr8 guides the nuclear RNase III enzyme, Drosha, towards it [87]. Drosha needs special terminal loops and regions to process pri-miRNA. Pre-miRNA has 5′ phosphate and 3′ hydroxy termini, and two- or three nucleotide 3′ single-stranded overhanging ends and the pre-miRNA is then transferred to cytoplasm. In the second step, Dicer makes a pair of cuts and each of these enzymes defines the ends of the mature miRNA [82]. Dicer cleavage’s outcome is a double stranded RNA of 22 nucleotides in length with overhangs on both ends. One of the strands of mature miRNA is degraded and the other strand is transferred into a member of the Argonaute (AGO) protein family [88], which has a crucial function in carrying out the silencing process based on miRNA. The function of miRNA at this moment is to guide the silencing complex to complementary regions on the target mRNA [89]. In other words, most miRNAs are transcribed from DNA sequences into primary miRNAs, then precursor miRNAs are processed from primary miRNAs, and eventually mature miRNAs are produced [90], and incorporated into the RNA-induced silencing complex (RISC) by binding to AGO proteins, and they function as post-transcriptional regulators by attaching themselves to the 3′-UTR of mRNAs [91]. What is more, miRNA genes are also controlled epigenetically by DNA methylation and modifications of histones [92].

Several non-canonical pathways for the biogenesis of miRNA have been reported alongside the canonical pathways [93]. They are similar to canonical miRNAs in terms of structure and functionality; however, it has been discovered that they mature in different ways, frequently skipping one or more steps of the traditional canonical biogenesis process [87]. Nonetheless, a shared characteristic of all these pathways is that the intermediate precursor is subjected to cleavage by Dicer [93]. Dicer’s absence results in the inability to generate the majority of functional miRNAs. The participation of Drosha and Dgcr8 is necessary for the processing of canonical miRNAs, but non-canonical miRNA biogenesis remains feasible even in their absence. To be precise, erasing Drosha or Dgcr8 leads to a total loss of canonical miRNA production, whereas the creation of non-canonical miRNAs remains unaltered [87]. An exemplar of non-canonical pathway is the processing of miR-451, which has been experimentally verified to evade Dicer cleavage and undergo cleavage by Argonaute-2 (Ago2) instead [93].

Non-canonical miRNAs were discovered to originate from a variety of sources, including introns, snoRNAs, endogenous shRNAs, and tRNAs. They have been discovered to participate in a number of biological processes, including stem cell growth and immunological responses [87]. On the other hand, efforts have been undertaken to determine the functions of non-canonical miRNAs in physiological processes or the onset of pathological conditions. Convincing evidence has established a correlation between the disturbance of non-canonical miRNAs and a range of diseases [94].

The miRtron pathway was the initial non-canonical pathway to be identified [87]. MiRtrons are a group of non-canonical pri-miRNAs that are located within the introns of coding genes [91]. This alternate pathway operates in the cytoplasm with the aid of Dicer, without requiring the assistance of the Drosha/Dgcr8 complex in the nucleus to generate pre-miRNAs [87]. Many of the animal miRNAs, roughly 40%, are encoded within the introns of genes that code for proteins, which means that a considerable number of pre-miRNA transcripts function as both pre-mRNAs and pri-miRNAs at the same time [95]. Upon scrutinizing the small RNA sequencing data in Drosophila, it has been observed that small RNAs arise from short introns of pre-miRNA size that possess the potential to form hairpins [95]. The process of miRtron maturation commences with the splicing and debranching of intron lariat, which stands in contrast to the splicing-independent biogenesis of canonical miRNAs that are located within introns. Specifically, in the case of canonical miRNAs, the cleavage of the precursor by the enzyme Drosha occurs before the host intron undergoes splicing [96]. Moreover, hairpin length and GC content can be used as criteria to distinguish between canonical miRNAs and miRtrons [97]. The discovery of the miRtron pathway has revealed not only a universal approach to miRNA biogenesis in various animal species, but also established a model for exploring alternative miRNA pathways. As a result, this area of research has proven to be fruitful, yielding a range of drosha-independent pathways that utilize different aspects of snoRNA or tRNA processing for miRNA biogenesis [98]. Furthermore, non-canonical miRNAs can also originate from transfer RNAs (tRNAs) [99]. By cleaving the stem of tRNA’s clover-leaf structure, DICER or Angiogenin can produce tRNA-derived RNA fragments [91].

Regulating gene expression by miRNAs is dynamic, meaning that they regulate gene expression until reaching a steady condition [90]. The inhibition of mRNAs via miRNAs requires pairing the 3′-UTR of target mRNAs to miRNAs. The optimum nucleotides to be continuously paired is 2 to 8 nucleotides. The inhibition occurs in miRNA-induced silencing complex (miRISC), which has components including AGO proteins and glycine-tryptophan protein of 182 kDa (GW182) proteins. GW182 has interactions with both AGOs and poly (A) binding protein (PABP) that these interactions call up the deadenylases CCR4 and CAF1 and targets mRNA [100].

The primary function of miRNA in the human body is the control of gene expression through canonical and non-canonical mechanisms for regulating transcription and translation as well as influencing the degradation of mRNA [101]. According to the canonical process, the target mRNA’s 3′-UTR is where the miRISC complex, which contains the miRNA guide strand, binds to exert its effect [102]. Based on the miRNA seed sequence, which is the first 2–7 nucleotides from the 5′ ends, this process occurs. mRNA deadenylation, translation inhibition, and destruction are the next steps [103–105]. Since their chains are not always completely complementary, about 60% of interactions between the miRISC complex and mRNA in human cells are non-canonical [106]. This raises the prospect that a large number of biological processes may be governed by this relationship since one miRNA may be able to target several mRNAs while at the same time, one mRNA may include many miRNA binding sites [102]. Intercellular signaling is one of miRNA’s additional significant functions. Despite the fact that the majority of miRNAs are found inside the cells, a significant number of them move outside of them and may be identified in body fluids [107–111]. These so-called circulating miRNAs are released into bodily fluids such as blood, urine, saliva, seminal fluid, and breast milk [30, 34] as a result of tissue destruction, apoptosis, and necrosis [101], or by active transit in micro vesicles and exosomes, or by binding to a protein [112, 113]. The existence of exogenous miRNA in the blood of healthy patients has also been challenged [114, 115], with its origin attributed to bacteria, food, and fungus, predominantly from the gut [102]. About 10% of the circulating miRNAs are secreted in exosomes, according to previous research, while the remaining 90% form complexes with proteins like high density lipoprotein (HDL), nucleophosmin 1 (NPM 1), and Ago2 [113, 116, 117]. This type of packaging is necessary to stop miRNA from being digested by RNases that are known to exist in body fluids [118]. It is also taken into consideration that these exogenous miRNAs may have pathogenic effects.

## MiRNAs in the immune system and inflammation

When a host is exposed to a virus, the immune system first detects alloantigen and triggers the activity of the signaling pathway associated with the immune response. However, the virus may escape the immune system, leading to the induction of cellular processes deficiency in the target cell; however, to avoid extreme inflammatory reactions, the immune system requires self-control mechanisms as well [119–121]. In the early stages of SARS-CoV-2 infection, the virus causes severe lymphopenia by targeting T lymphocytes. Also, the induced inflammatory response, such as the innate and adaptive immune response, leads to further induction of lymphocyte apoptosis [122]. On the other hand, the increase in systemic inflammation caused by cytokine storms, such as the secretion of interleukin-6 (IL-6), IL-2, and IL-7, leads to damage to other organs [121, 123, 124].

SARS-CoV-2 encodes its own miRNAs that can enter the host cell and, because of their small size, are not recognized by the immune system as alloantigen. Also, they inhibit and disrupt the immune system by targeting the immune system’s genes [119]. In addition, these miRNAs increase infection and virus replication by binding to functional regions of the virus’s genomic RNA. Besides, the RNA genome of SARS-CoV-2 inhibits host miRNA’s activity, disrupting the host’s immune system response. Also, some miRNAs activate immune-related signaling proteins to defend against viral infection. For example, in HIV-1-infected macrophages, an increase in miR-221 and miR-222 reduces CD4 expression and limits the spread and production of HIV-1 [125].

Furthermore, miRNAs expressed by SARS-CoV-2 affect the host immune system and the inflammatory response during viral infection. According to computational identification of SARS-CoV-2-encoded miRNAs, we can mention binding of miR385-3p to 5′-UTR TGFBR3 as a major immunosuppressant, binding of MR147-5p to two inflammatory proteins such as CXCL16 and ARRB2, binding of MR66-3p to tumor necrosis factor (TNF)-activator, binding of miR147-3p to TMPRSS2 enhancer, binding of miR198-3p to interferon (IFN)-related genes, and finally binding of miR359-5p and miR328-5p to proteins associated with viral infection such as MYH9 and RARA [31]. Interestingly, apoptosis-related proteins, including CHAC1 and RAD9A, are regulated by MD2-5p and miR147-3p, which are implicated in the apoptosis process caused by host cell infection [31]. Mechanically, the beginning of clinical manifestations of COVID-19 disease may be attributed to viral infection inducing cellular pathways, the release of inflammatory cytokines such as IL-6, hyperactivity of the JAK/STAT pathway, and the Akt/mTOR/HIF-1 signaling pathway [126–128].

Toll-like receptors (TLR)-like receptors detect viral antigens, which stimulate the innate immune response and modify the expression of miRNAs, regulating, increasing, or inhibiting the immune response and secreting cytokines [32]. In addition, SARS-CoV-2-induced infection depends on host miRNAs, which modulate immune response and inflammation. MiR-146a is the first miRNA implicated in regulating the innate immune response and inflammation following viral infection. In other words, miR-146a is produced in response to inflammation, which leads to a decrease in inflammatory responses. To that point, nuclear factor κB (NF-κB) is activated in chronic inflammation, which increases the production of miR-146a and subsequently negatively affects inflammation by impacting IL-1 and TNF-α receptors [129]. In this context, reduced miR-146a expression in the circulation induces inflammatory disorders in target organs such as the lungs, heart, brain, skin, and underlying vascular disease or autoimmune. Moreover, miR-146a reduces NF-κB signaling in NK cells by targeting the IL-1 receptor-associated kinase 1 (IRAK1) and TNF receptor-associated factor 6 (TRAF6) proteins, suppressing IFN-α production. IFN-α is a necessary cytokine generated by NK cells that causes the eradication of virus-infected and cancer cells [32]. It has been shown that miR-146a suppresses NK cells by overstimulating them with pro-inflammatory cytokines such as IL-12 and IL-18. In other words, miR-146a induction is appropriate for fine-tuning the activity of NK cells within an acceptable range [130, 131].

**Fig. 1:**
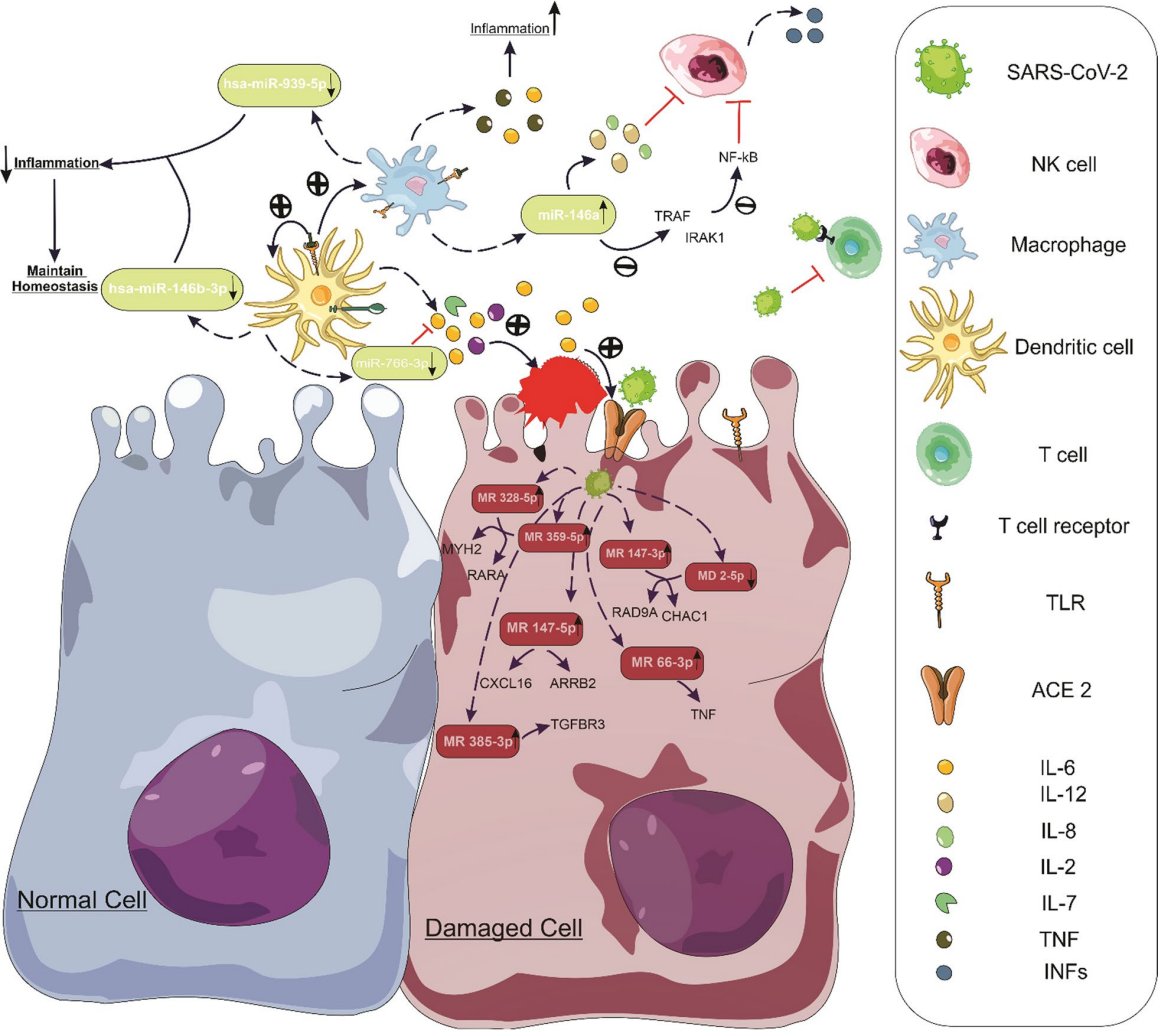
General mechanism of human and viral miRNAs function in target organs’ inflammation induced by SARS-CoV-2 infection. Following the entry of the virus into the target cell and transcription of viral miRNAs (red), these miRNAs increase cell inflammation and impair cell function by binding to cellular proteins. In addition, overexpression of immune cells leads to excessive secretion of inflammatory cytokines. While, the expression of host miRNAs (light green) by regulating the immune cells’ function and the inflammatory cytokines secretion can in turn reduce the inflammation and lead to the maintenance of cell homeostasis

MiR-766-3p, an anti-inflammatory miRNA, has been found to suppress IL-6 secretion in TNF-α stimulated MH7a cell lines; decreased TNF-α during COVID-19 infection may lead to increased IL-6 production [33]. According to Farr RJ et al. study in 2021, miR-31-5p, miR-27a-5p, and miR-766-3p also performed poorly in individuals with COVID-19 [34]. They also demonstrated that during SARS-CoV-2 infection, miR-423-5p, miR-23a-3p, and miR-195-5p are produced in the early stage of infection, which may be distinguished from influenza infection [34]. Also, two host miRNAs, hsa-miR-939-5p and hsa-miR-146b-3p, maintain homeostasis by reducing inflammation; however, they cannot attach to the mutant strain’s genome [31]. These two miRNAs, which are hijacked by the viral genome, are implicated in immune response pathways during viral infection. The following study showed that viral infection modifies the expression of host miRNAs, which influences the immune response and may lead to immune evasion of virus and dysfunction. In other words, the SARS-CoV-2-encoded miRNA is also related to cytoskeleton dynamics, which enhance both viral envision and trafficking through into the cell and attacked to the target organs. Besides, viral miRNAs can decrease the expression of genes involved in both apoptosis and the fatty acid metabolism process by binding to the 3′-UTR region in the host genome [31]. The disorders are generally caused by the aberrant production of miRNA in the cell or blood. In this context, miRNAs have been developed as a diagnostic and prognostic indication in various conditions, including diabetes, vascular disorders, viral infections, and malignancies [132–136]. Indeed, miRNAs, as regulators of gene expression, regulate various genes and cover conventional coding RNA (transcription factor, enzyme, etc.), non-coding RNA (ncRNA), and mitochondrial transcripts [123] (Fig. 1).

## The potential role of miRNAs as biomarkers

The medical community today recognizes biomarkers as distinct molecules, frequently proteins, that may be found in the body’s various fluids utilizing specific methods in laboratory environments. Troponin for the diagnosis of myocardial infarction, carcinoembryonic antigen (CAE) for various cancers, aminotransferases ALT and AST for liver diseases, and prostate-specific antigen (PSA) for the diagnosis and prognosis of prostate cancer are the best-known protein biomarkers detectable in blood [137]. Nevertheless, in recent years, the lack of clinically significant proteins, the complexity of their structures, and the difficulty in developing precise detection methods have made it time-consuming and expensive to identify new and improved protein biomarkers [138].

For roughly six decades, it has been recognized that unbound nucleic acids circulate within the bloodstream. Conversely, the detection of tumor-associated DNA and RNA within the plasma of individuals with cancer is now a standard diagnostic practice [138]. The notion that RNA molecules extracted from blood samples could not be utilized as biomarkers due to the presence of elevated levels of nucleases in plasma was once widely accepted. However, subsequent findings that miRNAs remain stable in fixed tissue samples have discredited this belief. [138].

MiRNAs were first identified as cancer biomarkers when Lawrie et al. utilized them to investigate diffuse large B-cell lymphoma in patient blood in 2008. Since then, literature has discussed miRNAs’ potential to be used as biomarkers for a variety of disorders [139]. However, because the study of miRNAs as biomarkers is still in its early stages, the results are currently generally unreplicable. There are many differences between the results of several group of scientists who analyzed the same tumors [140]. Standardized procedures must be created for both the early phases of the procedure, such as sample collection, transport, and storage, as well as data analysis for the variety of technical approaches employed, in order to effectively address this problem [138].

### The techniques for miRNAs identification

Research into molecular testing has focused on developing efficient and affordable methods for detecting miRNAs involved in some of the most prevalent diseases in the world, as well as trying to identify new such molecules associated with these pathologies [138]. This work has been done in order to aid the diagnostic process and optimize strategies for treatment. The field of miRNAs is still relatively recent, so characteristics like detection limits, the range of concentrations in bodily fluids, and modulation depending on different factors (age, gender, health/disease) are not yet completely understood. This has made the pursuit of this goal rather difficult [141].

Quantitative reverse transcriptase polymerase chain reaction (RT-qPCR) is the gold standard for miRNA quantification [141–143]. The primary PCR method employed in research has the advantages of high sensitivity and specificity rates and is based on the TaqMan microRNA test with stem-loop reverse transcription (RT) [144]. Another popular approach for evaluating miRNAs quantitatively is northern blot hybridization [145].

The identical goal of identifying the miRNAs is also accomplished using two other techniques. In situ hybridization, also known as ISH, is a method that compares the expression of miRNAs in diverse cells by using radioactive, fluorescent, or dioxygenin probes to bind the target RNA [146].

### Advantages and disadvantages of miRNA

This new class of miRNAs has a number of benefits that make them potential candidates for biomarkers in many different kinds of diseases. As previously stated, the ideal biomarker needs to be accessible, and miRNAs satisfy this need by being readily retrieved from blood, urine, and other body fluids using liquid biopsies. It has been used in numerous studies for the differentiation of the cancer stages [147] and even for the measurement of therapy responsiveness [148]. It also has a high degree of sensitivity for the tissue or cell type of source and it is susceptible in the way that it varies according to the development of the disease. Furthermore, compared to developing new antibodies for protein biomarkers, the development of new assays for the detection of nucleic acids takes less time and is less expensive. Another benefit of miRNAs is that they have the potential to be employed as multi-marker models for precise diagnosis, directed therapy, and assessing treatment responsiveness. While utilizing a large number of protein markers may be costly and time-consuming, using multi-marker panels made up of a large number of miRNAs may offer a non-invasive method for identifying diseases and advancement forecasting. For instance, finding the lupus nephritis urine miRNA signature has aided in the early identification of renal fibrosis [149]. This is crucial in diseases like cancer, which are very heterogeneous and would benefit from a multi-marker approach. This amount of improvement in breast cancer detection accuracy has already been demonstrated by a nine-miRNA multi-marker panel for breast carcinoma [150]. On the other hand, the core requirements and the most crucial assessment criteria for circulating miRNAs as diagnostic or prognostic biomarkers for clinical use are high sensitivity and specification. Since their levels in patients and healthy controls overlapped, single miRNA molecules did not easily satisfy the criteria for many candidate miRNA biomarkers. This finding showed that there should be significant individual variation in the amount of a relevant miRNA biomarker, increasing the risk of a false-positive or false-negative diagnosis [138].

Multiple cell-free miRNAs showed altered pattern of expression in a range of carcinomas rather than a single malignancy type. For instances, miR-21-5p, miR-155-5p, and miR-210-3p, all of which have been associated with various cancers including NSCLC [151–155], breast cancer, and colorectal cancer [156].

## Role of miRNAs as promising potential biomarker for COVID-19

MiRNAs can act as biomarkers for many diseases, especially infectious diseases [90, 157, 158]. Indeed, they are associated with viral infections, thus miRNA dysregulation can be considered as biomarkers of the disease. It means that they can be thought of not only as potential therapeutic alternatives, but also efficient in the diagnosis and prognosis [159]. Assessing the circulating miR-29c, miR-30c, miR-193a-5p and miR-885-5p may be useful in diagnosing HTLV-1 infection. Moreover, miR-29 may be considered as disease progression biomarker in patients with chronic hepatitis B virus infection [159].

There are many miRNAs that can be considered as potential biomarkers for COVID-19 diagnosis [157], some of them will be mentioned. MiR-21-5p, miR-146a, miR-126-3p, miR-144, and miR-155 are miRNAs discussed to be efficient as COVID-19 biomarkers. MiR-21-5p expression is less in COVID-19 patients than healthy human beings, thus being a good choice as a biomarker. Furthermore, it was more specific for SARS-CoV-2 infection than other respiratory infections and it is associated with inflammation as well. Likewise, miR-144 is expressed less in COVID-19 patients than in healthy human beings, making it a biomarker target as well. In contrast, miR-155 expression is higher in COVID-19 patients and is associated with lung injury [159].

Other miRNAs including plasma miR-19a-3p, miR-19b-3p, and miR-92a-3p are thought to be as biomarkers as well [157]. MiRNAs can be assessed as biomarkers in different stages of the disease including early stages and late stages [160]. For instance, miR-19a-3p, miR-19b-3p, and miR-92a-3p are expressed differently in the early phase of infection in COVID-19 patients compared to healthy ones [157]. MiR-155 expression is higher in patients with mild COVID-19, compared to severe COVID-19, thus making it an appropriate disease progression biomarker. MiR-146a and miR-126-3p are biomarkers of a pro-inflammation, and are downregulated in severe COVID-19. Recognizing efficient COVID-19 biomarkers can be extremely useful for applying precise therapy [159].

## The role of miRNAs in lung disorders AND inflammation associated with COVID-19

Lung is one of the organs SARS-CoV-2 infects and lung edema is one of the important manifestations of the disease, which 30% of COVID-19 patients develop it due to infection. Immune cells and some molecules are involved in this inflammation [161]. To mention the importance of lung in COVID-19, lung produces half of total platelets in the body, which have receptors for pathogens [162].

In COVID-19, the innate and adaptive immune system is activated including dendritic cells (DCs), macrophages, and T cells, result in the secretion of type I IFN cytokines such as interleukin 6 (IL-6) and TNF that all the above cause ACE2 expression [23, 163]. COVID-19 patients with lung symptoms have more leukocytes and IL-6, which causes exacerbated inflammatory responses, cytokine storm, and organ failure accordingly. There are multiple miRNAs involved in COVID-19; however, let-7a-3p, miR-135b-5p, miR-16–2-3p, and miR1275 are downregulated and miR-155-3p and miR-139-5p are upregulated during the infection [5]. In addition, some miRNAs have been recognized as having great association with inflammation and immune system including miR-155. Not only the higher rate of miR-155 is related to more severe inflammatory symptoms, but also can be beneficial in distinguishing COVID-19 from influenza [26, 35]. miRNAs are immune responses’ regulators in lung infection caused by SARS-CoV-2 and miRNA dysregulation have been reported in COVID-19 patients with lung symptoms. These dysregulations include the downregulation of the miR-320 family, miR-320a, miR-320b and miR-320c, and the upregulation of miR-320a and miR-320b [36]. Downregulation of miR-320 effects nuclear factor-erythroid factor 2-related factor 2 (Nrf2) expression and the upregulation of it effects TGF-β signaling pathways, Hippo signaling pathways, and inflammation in lung accordingly. The miR-320 family is downregulated in inflammatory diseases including COVID-19 [36]. Cytokine storm happening in lung is correlated with the activation of the NF-κB pathway [164].

On the other hand, the ACE2 is expressed commonly in lung alveolar cells and is the receptor of SARS-CoV-2, and it has been shown that ACE2 expression is related to lung inflammation [37]. Inhibiting the receptor of virus may be a suitable approach to treat COVID-19 patients [5]. In this way, miRNAs can participate in many biological steps in lung during viral infection. Nevertheless, it has been reported that only a few miRNAs are expressed in lung [27]. miR-27b influence the expression of ACE2 receptor, and miR-200b-3p, miR-200 c-3p, and miR-429 are able to downregulate ACE2, thus having a role in lung symptoms of COVID-19 patients [5]. Moreover, miR-143-3p can regulate the expression of ACE2 (inhibit ACE2 expression), but more experiments are needed to find out more about the association of miR-143 to immune system responses [37]. On the other hand, hsa-miR-98-5p can bind to 3′ UTR of TMPRSS2 gene transcription in lung endothelial cells. The hsa-let-7e/hsa-mir-125a and hsa-mir-141/hsa-miR-200 miRNA families can suppress ACE2/TMPRSS2 gene transcription as well [27].

Other miRNAs may involve in lung damage, including miR-21, miR-125b, miR-199a, miR-211, miR-138, miR-211, miR-146a, and miR-146b [27]. miR-1307-3p can prevent genome replication of SARS-CoV-2 by regulating BCL2 [38]. miR-1307-3p is also engaged in TGF-β signaling pathways in COVID-19 [5]. miR-1307 can influence TGF-β gene and consequently play a key role in lung fibrosis [39]. In contrast, miR-15b is involved in lung cancer and also has been discovered that it plays a role in SARS-CoV-2 infection. miR-30c and miR-15b are miRNAs participating in lung cancer, and miR-15b is downregulated in the lung of infected hamsters with SARS-CoV-2 [165]. miR-26a-5p, miR-29b-3p, and miR-34a-5p are downregulated in the COVID-19 patients’ lungs, thus considering them as therapeutic approaches in COVID-19 treatment [73]. Moreover, hsa-miR-17, capable of binding to viral proteins, is downregulated in viral infections [40], and miR-26a-5p, miR-29b-3p, and miR-34a-5p are downregulated in the lungs of COVID-19 patients [39]. Furthermore, miRNA MD241-3P is a viral miRNA capable of inhibiting morphogenetic protein receptor type 2 (BMPR2) gene and causing lung problems [27].

Overall, mentioned miRNAs such as miR-26a-5p, miR-29b-3p, and miR-34a-5p as well as viral microRNAs are associated with endothelial dysfunction and inflammatory response in patients with SARS-CoV-2 infection as well as the development of severe lung damage and immunothrombosis.

## The role of miRNAs in heart disorders AND inflammation associated with COVID-19

The link between SARS-CoV-2 virus infection and cardiovascular diseases is now evident, and individuals with COVID-19 have been documented to have previously severe illnesses [166, 167]. The arteries are impacted in the acute phases of SARS-CoV-2 infection, leading to endothelial cell (EC) activation, endothelitis, vascular permeability, and thrombosis in individuals with severe COVID-19 [168]. In other words, because viral receptors, including ACE2 and acetylated sialic acid residues are widespread on the surface of vascular ECs, they induce cardiovascular disorders [169–172]. However, the SARS-CoV-2 virus’s potentially infect and replicate in the ECs region is restricted; secondary processes such as the immune response cascade can stimulate endothelial cell disorders [173, 174]. Therefore, with severe COVID-19 illness, cardiovascular involvement might range from venous and arterial thrombosis to arrhythmia and myocardial infarction [175].

Furthermore, it has been discovered that insulin inactivity enhances adaptive cellular and thrombotic stress, contributing to cardiovascular problems in severe COVID-19 infections [23]. Following SARS-CoV-2 infection of cardiomyocytes, cytotoxic effects have been seen in the expression of genes involved in cellular metabolism and immune response. Pro-inflammatory cytokines are also effective against cardiac arrhythmias, hypotension, reduced blood flow, coagulation, and microvascular clotting, which causes small arteries to become plugged [23]. Numerous studies have found that several miRNAs are involved in cardiovascular and metabolic abnormalities, resulting in involvement and disorder in critically ill patients. For example, miRNAs such as miR-26b-5p and miR-200c-3p that target ACE2 expression in COVID-19 infection may affect the severity of cardiovascular diseases (CVD) [41–43].

It has been shown that ACE2 mRNA and protein expression is inversely related to the expression of miR-200c. In other words, this miRNA reduces the expression of ACE2 in cardiomyocytes by targeting the 3′-UTR region of its mRNA [41]. On the other hand, TMPRSS2 expression is inhibited by miR-98-5p and binding to 3′-UTR in human endothelial cells [44]. In this regard, during the bioinformatics study of Narsisyan et al. it was found that suppressing the transcription of let-7e/miR-125a and miR-141/miR-200 by targeting the 3′-UTR region, the expression of ACE2 and TMPRSS2 intensity decreases [176]. They also reported that the expression of these two miRNAs is essential for the function and expression of ACE2 and TMPRSS2 receptors. Also, miR-98 may play a crucial role in regulating TMPRSS2 function in COVID-19 disease [44, 45]. MiRNAs such as miR-21-5p, miR-155-5p, and miR-214 are linked to the modulation of pro-inflammatory cytokine release in the heart, whereas miR-125b and miR-223-3p are associated with the insulin signaling pathway and heart function [23, 46, 55, 177]. As a result, these miRNAs diminish endothelial cell damage induced by excessive glucose levels and inflammation. Disorders in the miR-590-3p modulation, which acts as a regulator of IL-6 and TNF-α production, have also been linked to myocarditis and heart failure. Thus, miR-590-3p expression through NF-κB regulation improves heart function [48]. Furthermore, miR-146a decreases inflammation and cardiac injury by adversely regulating the NF-κB pathway. Because miR-146a is not highly regulated in diabetics, its expression in COVID-19 reduces sepsis-induced cardiac failure or diabetes mellitus [23]. miR-30e-3p is one of the miRNAs implicated in cardiovascular issues induced by SARS-CoV-2, and it suppresses virus replication by binding to the complement region in the viral genome [49].

In a study by Tacke F et al. [178] it was found that circulating miR-133a levels in people with cardiopulmonary disorders were significantly higher. They also showed that neutrophil counts were inversely related to miR-133a expression. In addition, myeloperoxidase (MPO), as a marker of neutrophil activation in cardiovascular disease, endothelial cell apoptosis, and matrix metalloproteases, was directly related to miR-133a levels [50–52]. In other words, neutrophils can be considered as a secondary source of circulating miR-133a due to neutrophil degranulation and extravasation results in myocyte damage [179, 180].

Furthermore, it has been observed that the expression of miR-423-5p, miR-23a-3p, and miR-195-5p is dramatically enhanced in cardiovascular disorders [53, 54]. On the other hand, reduced expression of these miRNAs leads to a reduction in severe COVID-19 infection in CVD patients. Because of the positive relationship between elevated levels of miR-153, miR-208a-3p, and miR-375 with heart disorders and myocardial damage [55–57]. It is worth noting that miR-208a and miR-375 are significantly expressed in dilated cardiomyopathy patients and promote apoptosis in ischemic cardiomyocytes; therefore, diminishing their expression improves heart disease treatment [55–57].

In general, anti-miRNA therapy and manipulation of particular miRNAs against COVID-19 can reduce symptoms and improve cardiac function in individuals with severe COVID-19.

## The role of miRNAs in brain disorders AND inflammation associated with COVID-19

As mentioned, severe infection with the SARS-CoV-2 virus results in a systemic disease that leads to inflammation and neurological symptoms and increases heart failure and pulmonary disorders. It is reported that 30% to 60% of COVID-19 patients have neurological symptoms [181]. Neurological symptoms include loss of smell, change in taste, loss of coordination, headache and nausea, dizziness, intermittent loss or disturbance of consciousness, acceleration or exacerbation of pre-existing cognitive impairments, direct adverse effects of an aggravated immune response, more responses, unusual inflammation, increased vascular inflammation, the brain and spinal cord inflammation, and atherogenesis, exacerbation or new induction of inflammatory neuronal destruction, loss of respiratory control, and progressive cognitive impairment including encephalitis, stroke, seizures, encephalopathy, and diffuse encephalomyelitis [182–186].

ACE2 is significantly expressed in different areas of the brain. The highest expression of ACE2 in the brain is in the respiratory centers of the brain, pons, and medulla oblongata; as a result, it can lead to abnormal breathing and pulmonary manifestations such as shortness of breath [61]. In theory, infection with SARS-CoV-2 causes the activity of immune cells, resulting in the release of various cytokines, chemokines, and antibodies. The disorder occurs in the CNS by the nose or eyes, and virus particles initially reach the olfactory bulb, then the brainstem, and ultimately all brain regions [187]. In addition, SARS-CoV-2 can systematically target nerve cells through blood vessels from the blood–brain barrier (BBB). Systemic infection has been reported due to increased secretion of pro-inflammatory agents into the bloodstream and cytokine storms [188]. As a result, BBB permeability and subsequent transmission of SARS-CoV-2 to neurons are increased. Astrocytes and microglia become active after the virus targets CNS. The immune response of astrocytes and microglia is also regulated by various miRNAs [187]. Interactions between miRNA and mRNA are fundamental in the progression of human neurological disorders [189–191].

Studies show that pro-neuroinflammatory miRNAs such as *mir-155, mir-27b,* and *mir-326*, and anti-neuroinflammatory miRNAs such as *mir-146a, mir-124,* and *mir-21* regulate inflammatory processes in the CNS [192]. Moreover, the relative expression of pro-neuroinflammatory miRNAs mentioned and their target mRNAs, such as peroxisome proliferator-activated receptors (PPARS), suppressor of cytokine signaling 1 (SOCS1), and CCAAT/enhancer-binding protein alpha (C/EBPA), increases and decreases during COVID-19 disease, respectively [193]. Furthermore, Lukiw WJ et al. showed that in individuals with COVID-19, the anti-neuroinflammatory miRNAs’ expression such as *mir-21, mir-124, and mir-146a*, and their target mRNAs, such as *IL-12p53, STAT3,* and *TRAF6*, decreased and increased, respectively [193].

For example, anti-inflammatory regulators such as SOCS1, SH2-containing inositol-5′-phosphatase 1 (SHIP1), CCAAT/enhancer-binding proteins (C/EBP), and IL-13R are affected by mir-155 [58–61]. In other words, mir-155, by stimulating transcription factor p53 and subsequently inducing c-Maf transcription factor, increases differentiation and inflammatory responses, thus inhibiting the suppression of anti-inflammatory signals and inducing neuroinflammation [62]. Mir-146a also acts as an anti-inflammatory regulator in nerve cells, microglia, and astrocytes. As mentioned, this miRNA is activated via the NF-κB-dependent TLR signaling pathway, so it acts as a kind of NF-κB pathway regulator by targeting MyD88 signaling components such as IRAK1 and TRAF6 [193, 194]. In addition, other inflammatory factors such as STAT-1, IFN regulatory factor 5 (IRF-5), CFH, and polarization of macrophages and microglia are altered by mir-146a [63–66]. mir-124, as an anti-inflammatory miRNA, plays an essential role in neuronal differentiation. This miRNA is highly expressed in microglia, unlike peripheral macrophages; as a result, it leads to an anti-inflammatory response through the M2 phenotype [67–69]. In other words, mir-124 has an anti-inflammatory role by reducing inflammatory factors and microglia activity.

mir-21 plays a vital role in different CNS cells, such as microglia and astrocytes, neurons, and oligodendrocytes [193]. Indeed, mir-21 is activated through the TLR signaling pathway as an anti-inflammatory regulator. This miRNA reduces TNF-α secretion in macrophages and microglia and increases IL-10 expression [70, 71]. Moreover, mir-27b, as a pro-inflammatory agent, acts by targeting an anti-inflammatory transcriptional activator PPAR, and it has been reported that the inflammatory signals are restricted by inhibiting mir-27b. This miRNA induces the secretion of inflammatory cytokines such as IL-6 and TNF-α [72]. Another type of pro-inflammatory miRNA, such as mir-326, affects the differentiation of Th17 cells [73].

MiRNAs generally affect neuronal signaling and the inflammatory and anti-inflammatory pathways cumulatively. For example, mir-146a and mir-21 target different components of the TLR/MyD88/NF-κB and JAK-STAT pathways, while mir-155, mir-27b, and mir-326 target SOCS1 and SHIP1 and activate the JAK-STAT pathway [59, 63, 65]. In addition, miRNAs such as mir-124, mir-21, and let-7 are presented in the exosomes, stimulating and regulating microglia cells to activate inflammatory signaling pathways [74]. Furthermore, it has been reported that the level of free miRNAs in the cells may decrease following their binding to the viral genome; as a result, people with COVID-19 infection are more likely to develop Alzheimer’s-like disease [195]. In this regard, it has been shown that miRNA-146a is involved in both Alzheimer’s and prion diseases [193]. However, whether induction of miRNA-146a following viral infection is a host defense mechanism or not and whether miRNA-146a inactivates ssRNAs such as SARS-CoV-2 is unclear [66, 196].

MiRNAs such as miR-130a-3p, miR-29b-3p, miR-27a-3p, miR-145-5p, and miR-200a-3p were shown to be distinct in the research by Prasad et al. [181] Also they indicated that miRNAs including miR-34a-5p, miR-200a-3p, and let-7a-5p interact with the SARS-CoV-2 virus. Moreover, a study conducted in 2021 found that CvmiR-5-5p derived from SARS-CoV-2 is likely one of the critical components in neurological diseases induced by SARS-CoV-2. In this context, anti-vmiRNA oligonucleotides suppression of CvmiR-5-5p in COVID-19 infection might be investigated as a therapy for neurological symptoms [20]. Finally, it should be emphasized that COVID-19 illness alters the relative expression of pro- and anti-neuroinflammatory miRNAs, lowering and rising, respectively. Additionally, micro RNAs regulate the immune response of astrocytes and microglia, which aggravates neurological disease indicators and patient complaints.

## The role of miRNAs in gastrointestinal disorders AND inflammation associated with COVID-19

Gastrointestinal symptoms such as diarrhea, abdominal pain, and vomiting are present in 2 to 10% of COVID-19 patients [197, 198]. According to a study, viral miRNA could target TMPRSS2, which together with ACE2 facilitates virus entrance in the gut [199]. Liu et al. also predicted that in the gut, a number of genes are affected by miRNAs. For example, miR-147-3p in the gut targets the TMPRSS2 enhancer and increases intestinal inflammation following infection with SARS-CoV-2 [31]. Besides, this miRNA, which is derived from the virus, also causes the virus to attack into the intestinal cells. In addition, the S gene of SARS-CoV-2-induced intestinal infection is affected by hsa-miR-4661-3p [31]. ACE2 is expressed highly in small intestine, colon, lung, and kidney and can consider these miRNAs involving in ACE2 expression as therapeutic options [75, 200]. miR-21 is able to inhibit ACE2 gene expression, and let-7a/ let-7d, miR-30a, miR-30c, miR-127, miR-194, miR-200c, miR-361, and miR-423 inhibit the expression of TMPRSS2. Both ACE2 and TMPRSS2 are needed for SARS-CoV-2 entry and the onset of infection and gastrointestinal symptoms of COVID-19 are due to targeting of TMPRSS2. Therefore recognizing miRNAs or anti-miRNAs targeting these enzymes will be a novel way of COVID-19 treatment [5, 75]. ACE2 expression is more extended in colon cancer patients. Thus, in patients with colon cancer, SARS-CoV-2 infection is more likely; which means that there may be an association between ACE2 expression and SARS-CoV-2 infection in colon [201].

Furthermore, in a study, miR-146, as one of the modulators of the immune response, was found in mice with larger spleens, and it leads to an increase in myeloid cells in the spleen and bone marrow, and eventually spontaneous increase in inflammation of the intestine [202]. Moreover, Farr RJ et al. observed that miR-31-5p is highly expressed in COVID-19 patients, and in turn modulates inflammation [34]. In endothelial cells, TNF-α induces miR-31-5p transcription and creates a negative feedback loop containing E-selectin to control inflammatory signals [203]. MiRNA-31-5p is also elevated in inflamed ulcerative colitis mucosa, where it suppresses the expression of signal transducer and activator of transcription 6 (STAT6), suppressor of cytokine signaling 1 (SOCS1), and eotaxin-3 (CCL26) via downregulating the IL-13 receptor α-1 (IL13Rα-1) gene [76]. In fact, miR-31-5p is expressed during COVID-19 to limit the excessive inflammatory response [34]. Remarkably, in animal studies of enterocolitis infected with SARS-CoV-2, the expression of miR-27a-5p was significantly increased [204]. As a result, increased expression of miR-31-5p and miR-27a-5p in COVID-19 patients indicates an increase in gastrointestinal infection by SARS-CoV-2 [205].

There are cellular miRNAs acting against SARS-CoV-2. Some of these miRNAs are downregulated in specific conditions i.e. miR-4684-3p and miR-518a-5p are downregulated in colorectal cancer patients and gastrointestinal stromal tumors, respectively [5]. One advantage of miRNAs is that miRNA-contained vaccines do not incapacitate neuronal cells, but they can act as immunogens in gastrointestinal pathway [39]. The furin enzyme is expressed in lung, liver, and small intestine. An isoform of this enzyme can help to cleavage the S protein of SARS-CoV-2. Its expression can be regulated by miR-20b, miR-19a, miR-19b-3p, and miR-106a [77].

Other miRNAs including miR-30c and miR-200c have a role in SARS-CoV-2 infections [75]. Furthermore, miR-219a and miR-30c are related to gastric cancer. Thus considering them a role in SARS-CoV-2 is not unexpected [165]. Moreover, three miRNAs including miR-3529-5p, miR-7–1-3p, and miR-548az-5p are proposed to be engaged in KEGG pathways which might be related to some of the COVID-19 symptoms [6]. hsa-miR-8066 and hsa-miR-5197-3p are critical in viral infections [27] and miR-4684-3p and miR-518a-5p are two main miRNAs decreased in COVID-19 [5]. Since viral miRNAs influence the expression of a number of genes, including genes related to ACE2 or TMPRSS2, during the progression of the COVID-19 disease, they typically cause an increase in the inflammatory response as well as an increase in clinical symptoms. Additionally, these microRNAs increase the virus absorption into intestinal cells, increasing the susceptibility of those cells to infection.

## The role of miRNAs in kidney disorders AND inflammation associated with COVID-19

As mentioned in the previous sections, ACE2 is expressed in the epithelial cells of the lung, heart, and kidney, hence the kidney can be considered as another target organ of the SARS-CoV-2 virus. Some of the important miRNAs having a role in COVID-19 renal symptoms include miR-18, miR-21, miR-216a, miR-15b-5p, miR-141, miR-181a, miR-200a, miR-421, and let-7 [23, 78]. Reports demonstrate that the downregulation of miR-141, miR-200a, and let-7 and upregulation of miR-216a can cause overexpression of TGF-β and consequently cause kidney fibrosis. Accordingly, by the inhibition of TGF-β signaling via miRNA mimics and inhibitors it is possible to decrease COVID-19 severity in kidney. miRNAs which their roles are regulating ACE2 can play a key role in COVID-19 as well [23, 78]. miR-181a is another miRNA, which is able to upregulate the signaling cascades of adaptive immunity and decrease COVID-19 renal symptoms [23, 78]. miR-18 and miR-125b are specifically expressed in the kidney and have important roles in enhancing ACE2 expression [206]. Therefore, applying anti-miR-18 may be a new therapeutic way to inhibit ACE2 expression by downregulating the beta-NADPH oxidase 2/reactive oxygen species (Nox2/ROS) pathway [206]. Also, miR-374a-3p and miR-15a-3p are miRNAs participating in kidney disorder, caused by SARS-CoV-2 infection [36]. MiRNAs associated with kidney disorders caused by COVID-19 have the ability to impact inflammatory pathways and increase the production of cytokines such as TGF-β. These actions contribute to the development of kidney fibrosis. Therefore, targeting these specific miRNAs could be considered a potential treatment approach to reduce symptoms and manage the effects of SARS-CoV-2 infection on the kidneys.

## The role of miRNAs in liver disorders AND inflammation associated with COVID-19

Primary liver dysfunction is related to poor outcomes in people with COVID-19. The evidence suggests that miR-122 is involved in the host response to liver and lung diseases during viral infections [81]. For example, miR-122 has drastically increased neutrophilic pulmonary inflammation after rhinovirus infections [207]. MiR-122 also plays a crucial role in the innate immune response in liver cells during viral infections [80]. Indeed, miR-122 promotes the production of type I and II IFNs in response to various viral nucleic acids [80]. In individuals with severe COVID-19 illness, the IFN response is inhibited, resulting in an inflammatory response and disease aggravation [208, 209]. Liver abnormalities in COVID-19 patients and low levels of liver-derived miRNA in non-survival and elderly patients may represent a distinguishing pathophysiological hallmark of COVID-19 compared to other critical disorders such as sepsis or acute respiratory distress syndrome [81]. In this regard, it has been reported that an increase in miR-122 levels in non-survival individuals during sepsis or acute respiratory distress syndrome may be due to liver disorders [210–212]. It has been observed that miR-122 serves as a prognostic marker for acetaminophen-induced liver failure [213]. Besides, miR-122 expression is directly related to the amount of plasma or serum proteins derived from the liver of COVID-19 patients [81]. Also, myocyte damage due to inflammation and hepatic acute phase response leads to the expression of both baseline levels of myocyte-derived miR-133a and liver-derived miR-122 in acute COVID-19 disease [81].

Viral miRNAs regulate genes involved in the actin secretion function and regulate the metabolic process of viral proteins in the liver. For instance, the adenosine deaminase acting on RNA enhancer, as an adenosine deaminase that acts on RNA and suppresses IFN system responses in viral infections, is regulated by MR198-3p [79].

The mechanism and outcome of different degrees of liver damage in COVID-19 patients are not fully understood. So the effect of miRNAs encoded by SARS-CoV-2 on enhancer genes that are expressed in the liver and the possible role of miRNA virus in COVID-19 liver injury has been investigated as a treatment strategy [31].

Some of the miRNAs we mentioned before, with their function, is summarized in Table1.

## Role of miRNAs in Diabetes associated with pancreatic during COVID-19

Studies demonstrate that diabetic patients are in danger of higher COVID-19 mortality than normal population. Glucose utilization, metabolism, and biological processes are not working properly in diabetic patients, which is not a good sign for cardiovascular systems. Studies have demonstrated that both diabetes and COVID-19 can worsen heart failure. The expression of specific miRNAs can serve as a useful biomarker for identifying heart failure [214].

MiRNAs can play a role as biomarkers for the severity of COVID-19 in patients with or without diabetes. In patients with both COVID-19 and diabetes, miRNA content can be an acceptable biomarker to distinguish the severity of the overall disease. MiRNAs can buffer immune metabolism in patients with diabetes and COVID-19. The connection between diabetes, metabolism, and the cardiovascular system is well-established. Two specific cardiovascular-related miRNAs, miR-15b-5p and miR-30e-3p, tend to decrease with age. These miRNAs may play a role in patients who have both diabetes and COVID-19 [214].

MiR-133a is a miRNA which can regulate ACE2 receptor function and it is available in the heart abundantly. The presence of miR-133a is crucial for maintaining optimal heart function in adults. When miR-133a is lost, it leads to cardiac hypertrophy and dysfunction. Interestingly, increasing the levels of miR-133a through overexpression does not have any harmful effects. Instead, it offers protection to the heart by preventing cardiac fibrosis resulting from pressure overload and preserving contractility in individuals affected by diabetes. As a result, miR-133a holds great potential as a research target to explore its involvement in heart failure among patients with diabetes and COVID-19 [214]. Other miRNAs including miR-1-3p, miR-34a-5p, miR-129–2-3p, miR-146a-5p, miR-16-5p, miR-101-3p, miR-671-5p, miR-155-5p, miR-124-3p, miR-20a-5p, and let-7b-5p have the same pathogenic implications among COVID-19 and diabetes [215].

Soluble ACE2 and miR-421 are diminished in diabetic patients. On the other word, miR-421, miR-3909, miR-212-5p, and miR-4677-3p are associated to ACE2 regulation. Accordingly, diabetic patients are more susceptible to COVID-19 than nondiabetic population [215]. In consequence, miRNAs can be considered as biomarkers and therapeutic alternatives in patients with both diabetes and COVID-19 [215]. In other words, miRNA regulates the expression of the ACE2 receptor in the host cells and approximately inhibits the immune system in patients with diabetes and COVID-19.

## Therapeutic approach and future perspective

Host miRNAs have been shown to play a crucial role in virus infection and reproduction. Likewise, viral miRNAs can cause significant alterations in host gene transcription. Interestingly, a number of miRNAs influence immune cell activity and host immunological homeostasis [23]. As a result, some chemistries can be regarded as good targets as antiviral medication, in order for miRNAs to function effectively in host–pathogen interactions. For instance, Davis et al.’s study aimed to find an appropriate antisense oligonucleotide that could effectively decrease miRNA activity or inhibit it. Few miRNAs have been given any previously identified functions, and antisense oligos are an easy way to block them. As the active molecule is a small RNA that will be difficult to target specifically without using Watson–Crick base pairing, antisense targeting may be the only method for therapeutically inhibiting miRNAs. For efficient functionalization and therapeutic targeting of miRNAs, it is therefore essential to determine the best chemistries for miRNA inhibition [216].

### Role of miRNA therapy in COVID-19

After transmission of SARS-CoV-2, the virus enters the host cell by binding to host cell receptors and fusing with the cell membrane. Specifically, membrane TMPRSS2 located in the host cell membrane cleaves the SARS-CoV-2 spike protein, allowing the virus to enter the host cell via ACE2 [23]. Therefore, targeting miRNAs involved in the expression of TMPRSS2 and ACE2 play an important role in therapeutic approaches during COVID-19 disease. For example, miRNAs miR-98-5p, let-7e-5p miR-7–5p, miR-92–3p, miR-214–3p, miR-511–3p, miR-4500, miR-6864–3p and let-7a-g/i suppress TMPRSS2 mRNA in human cells [5, 44, 217].

On the other hand, ACE2 is a type 1 integral membrane protein that carries amino acids and viral receptors [218, 219]. The miR-143 and miR-421 and the miR-27a/b and miR-145 expressions are inversely and directly related to the ACE2 expression, respectively [220, 221]. In addition, miR-106b-5p, miR-130a-3p, miR-141-3p, miR-200-3p, miR-300, miR-429, miR-2113 and miR-5197-3p have been identified as expression regulators of ACE2 [41, 222]. In this regard, targeting viral proteins and primary virus ligands by host miRNAs can be considered as a potential strategy for treating COVID-19. In this regard, Khan et al. reported that host miRNAs could target and bind to genomic regions such as ORF1ab, S, M, N, ORF3a, ORF7a, ORF8, 5’-UTR, and 3’-UTR. Therefore, inhibition of mRNA encoding the SARS-CoV-2 S protein could be considered as a potential candidate for treatment [223]. Moreover, in 2020, a study reported that miR-510-3p and miR-624-5p could target viral spike open reading frame (ORF) mRNA, while miR-624-5p is more effective in inhibiting spike RNA than miR-510-3p [224].

On the other hand, targeting nonstructural proteins and nucleocapsids can also be considered as a therapeutic strategy. In this regard, Khan et al., following the analysis of RNA-RNA interactions of the host miRNA genome and SARS-CoV-2 in 67 patients, reported that miR-506-3p, miR-6817-5p, and miR-12199 could interact with the SARS-CoV-2 mRNA nucleocapsid [223, 225]. Zhou et al. also showed that SARS-CoV-2 proliferation is significantly blocked by exosomes containing miR-2911 [226]. Furthermore, Nersisyan et al. showed that host miR-21-3p was inhibited following the infection by SARS-CoV-2 virus, thereby caused immune response delay and virus survival. MiR-21-3p is thought to bind to polypeptide 1a mRNA, a conserved region in human coronavirus [217]. Also, the expression of the histone deacetylase 8 (HDAC8) gene, as an essential modulator of chromatin structure, is reduced by miR-21-3p, inhibiting viral replication [217].

### Role of miRNA therapy in the immune system induced by SARS-CoV-2

Following infection with SARS-CoV-2 and cytokine storms development, the secretion of cytokines such as IL-1β, IL-2, IL-6, IL-7, IL-8, IL-9, IL-10, IL-17, G-CSF, GM-CSF, IFN-γ, and TNFα are increased. Besides, chemokines such as IFN-induced protein 10 (IP10), monocyte chemical adsorbent protein 1 (MCP1), macrophage inflammatory protein 1α (MIP1α), and MIP1β have also been identified in individuals with COVID-19 [78]. Consequently, miRNAs involved in the inflammatory response may be an applicable target in reducing the complications of SARS-CoV-2 infection.

MiR-136 induces the accumulation of IL-6 and IFN-β in mammalian cells by binding to the 3’-UTR of RIG-I’s transcript. Moreover, miRNAs such as miR-21-3p, miR-21-5p, and miR-155-5p significantly increase the secretion of IL-6 and IL-8 [227–229]. In contrast, IL-6, IL-8, and C–C motifs of the chemokine ligand 5 (CCL5) are reduced by miR-146a, miR-146b, and miR-200c while miR-17 and miR-93-5p suppress IL-8 expression [119, 230]. In addition, let-7 regulates TLR4 and subsequently affects NF-κB activity; also, this miRNA reduces the pro-inflammatory cytokines IL-6 and IL-8. It has been shown that let-7b regulates IFN type I, while miR-466i directly targets IFNα expression [23].

Following bioinformatics studies, it was shown that miR-5197-3p, which is essential for the TGF-β pathway, binds uniquely to the SARS-CoV-2 genome RNA and can be considered as a therapeutic strategy [231].Further to this, the TNF-α cytokine is regulated by miR-145, while miR-146a or miR-351-5p reduce the secretion of this pro-inflammatory cytokine [232].

Immune cells such as macrophages, neutrophils, and epithelial cells are activated by IL-1 secretion and increase the production of both T lymphocyte cytokines and Th2 cellular responses. In addition, Th17CD4 + lymphocytes exacerbate several autoimmune diseases by over-secreting the pro-inflammatory cytokine IL-17. As a result, reports suggest that targeting IL-1 and IL-17 can reduce cytokine storms and subsequent immune disorders in COVID-19 patients [233]. For example, the IL-1 signaling pathway can be regulated by miR-155, while secretion of cytokines IL-1, IL-6 and TNF-α is inversely related to miR-146a expression. In addition, IL-17 secretion has been shown to elevate following an increase in miR-142-5p, miR-21, miR-1266, and miR-29a, while, miR-146a, miR-182, miR-194, miR-15a/16 and miR-34a lead to a decrease in IL-17 secretion [234]. Therefore, miRNAs that regulate inflammatory and pro-inflammatory cytokines are speculated to be potential therapeutic targets for reducing the impairment of the immune response in the treatment of COVID-19.

## Conclusion

The interactions between host and virus-derived miRNAs regulate the severity of infection and the host’s defense response. Therefore, it is suggested that miRNAs may be a useful strategy for early identification and treatment of COVID-19 infection. By modulating miRNA expression, it may be possible to prevent disease development and reduce the severity of illness.

Overall, miRNAs provide several benefits in targeting and inactivating SARS-CoV-2, including the following:

1. Causes the establishment of a natural defensive mechanism or innate immune response based on ssRNA against SARS-CoV-2 viral invasion and replication [193].
2. Synthetic and chemically stabilized miRNAs can specifically target and breakdown viral RNA sequences, leading to potential therapeutic effects and the cessation of SARS-CoV-2 pathogenic activity [193].
3. It can potentially target and inactivate other infectious viruses with ssRNA genome, such as neurotrophic viruses [193].
4. They can be used to identify a particular stage of SARS-CoV-2 infection as biomarkers [235].
5. They are effective in pharmacological treatments and anti-SARS-CoV-2 therapeutic techniques [70].

## Data Availability

Not applicable.

## Abbreviations

SARS-CoV-2: Severe acute respiratory syndrome coronavirus 2
COVID-19: Coronavirus disease 2019
miRNA: MicroRNA
S: Spike
E: Envelope
M: Membrane
N: Nucleocapsid
ACE2: Angiotensin-converting enzyme 2
sACE2: Soluble ACE2
vmiRNA: Viral miRNA
UTR: Untranslated region
TMPRSS2: Transmembrane serine protease 2
RNase: Ribonuclease protein
IL: Interleukin
TNF-α: Tumor necrosis factor-α
pri-miRNA: Primary miRNA
pre-miRNA: Precursor miRNA
miRISC: MiRNA-induced silencing complex
AGO: Argonaute
IFN: Interferon
NF-κB: Nuclear factor κB
CVD: Cardiovascular diseases
BBB: Blood–brain barrier
ORF: Open reading frame
